# Incidence of human adenoviruses and *Hepatitis A virus* in the final effluent of selected wastewater treatment plants in Eastern Cape Province, South Africa

**DOI:** 10.1186/s12985-015-0327-z

**Published:** 2015-06-24

**Authors:** Olayinka Osuolale, Anthony Okoh

**Affiliations:** SA-MRC Microbial Water Quality Monitoring Centre, University of Fort Hare, Alice, 5700 South Africa; Applied and Environmental Microbiology Research Group, Department of Biochemistry and Microbiology, University of Fort Hare, Alice, 5700 South Africa

**Keywords:** Adenovirus, *Hepatitis A virus*, Wastewater, Eastern Cape, Effluent, Public health

## Abstract

**Background:**

Municipal effluent constitutes a large reservoir of human enteric viruses and bacteria. Contemporary monitoring practices rely on indicator bacteria, and do not test for viruses. Different viruses, including Norwalk-like viruses, *Hepatitis A virus* (HAV), adenoviruses, and rotaviruses, are important agents of illnesses in humans. The burden of disease caused by adenoviruses manifests as pneumonia, bronchiolitis, otitis media, conjunctivitis, and tonsillitis, whereas HAV infection can manifest as acute inflammatory diseases of the liver, fever, anorexia, malaise, nausea, and abdominal discomfort, followed by jaundice and dark urine. The public health implications of these viruses depend upon the physiological status of the wastewater microbial community.

**Methods:**

The occurrence of human adenovirus (HAdV) and HAV was determined in the final effluents of five wastewater treatment plants (WWTPs) in the Eastern Cape, South Africa, over 12 months (September 2012–August 2013). The viruses were detected with real-time PCR, and conventional PCR was used for serotyping.

**Results:**

Adenovirus was detected in effluent samples from all five WWTPs and in 64 % of the total samples, whereas HAV was not detected in any effluent sample. At WWPT-A, samples were collected from the final effluent tank (adenoviral concentrations ranged from 1.05 × 10^1^ to 1.10 × 10^4^ genome/L, with a 41.7 % detection rate) and the discharge point (adenoviral concentrations ranged between 1.2 × 10^1^ and 2.8 × 10^4^ genome/L, with a 54.5 % detection rate). At WWPT-B, HAdV was detected in 91.7 % of samples, with viral concentrations of 7.92 × 10^1^–2.37 × 10^5^ genome/L. The HAdV concentrations at WWPT-C were 5.32 × 10^1^–2.20 × 10^5^ genome/L, and the detection rate was 75 %. The adenoviral concentrations at WWPT-D were 1.23 × 10^3^–1.05 × 10^4^ genome/L, and the detection rate was 66.7 %. At WWPT-E, the viral concentrations were 1.08 × 10^1^–5.16 × 10^4^ genome/L, and the detection rate was 54.5 %. Characterization of the adenoviruses revealed HAdV serotypes 2 (1.4 %) and 41 (7.1 %), in species C and F, respectively.

**Conclusions:**

This study is the first to report the prevalence of HAdV in the final effluents of WWTPs in the Eastern Cape, South Africa. The adenoviral detection rates indicate the potential contamination of the environment, with adverse effects on public health.

## Introduction

Human adenoviruses are ubiquitous in the environment and humans are the only reservoir for them. They are excreted in large numbers in human feces. Although adenoviruses have been reported to infect a variety of animals, they are more reported in humans to be highly specific to them. The viruses persist wherever the environment has been polluted by human feces or sewage [1–3]. Therefore, in natural aquatic environments, the incidence of human adenovirus is probably attributable to contamination with untreated or inefficiently treated sewage [3]. Various variants of adenovirus have been identified, and over 50 serotypes are known [4] throughout the world [5, 6]. Human adenovirus (HAdV) has been implicated in infections causing gastroenteritis, conjunctivitis, and respiratory diseases [7], and chronic systemic infections in immunosuppressed individuals [8, 9].

*Hepatitis A virus* (HAV) is the principal cause of acute hepatitis and is currently recognized as one of the most important human food-borne pathogens in the world. It is responsible for around half the cases of hepatitis diagnosed worldwide. HAV has a worldwide distribution and its presence varies between regions and localities [10]. It can be transmitted via the fecal–oral route, either by person-to-person contact or by the ingestion of contaminated water and food, especially in endemic areas [11]. Like HAdV, humans are the only known reservoir for HAV [1].

Municipal effluent constitutes a large reservoir of human enteric viruses and bacteria [12]. Contemporary monitoring practices are based on indicator bacteria, and do not test for viruses. Various viruses, including the Norwalk-like viruses, *Hepatitis A virus*, adenoviruses, and rotaviruses, are important agents of illnesses in humans [13]. Their public health implications depend upon the physiological status of the microbial communities in wastewater [12]. The occurrence of HAdV and HAV in raw water sources reflects the epidemiological features of the environment, including disease outbreaks in particular communities [1]. Globally, adenoviruses have been detected in various types of water, including swimming pools, oceans, river water, and wastewater [6], and they contaminate the surface waters when pollution enters a water body [14, 15]. HAV and HAdV have been detected in raw and treated water, dams, rivers, and rivers receiving effluent discharges in South Africa [16–20]. Screening stool samples in surveillance programs has confirmed the presence of adenoviral [21, 22] and hepatitis A virus antigens in the samples [23, 24]. The viruses have been detected in the stools of patients in South Africa [19, 25], and gastroenteritis in infants, toddlers, and children has been attributed to them [26, 27].

The epidemiological importance of viruses as water-borne pathogens continues to receive attention, and wastewater is a significant object of research because the diversity of viruses excreted in human waste is high [28]. Human feces and urine can contain enormous amounts of enteric viruses excreted from infected individuals. Therefore, wastewater is one of the major concentrated sources of these viruses [29]. The detection of water-borne viruses is very important to public health, in the deterrence of illness and in response to outbreaks. Although these viruses are considered important, insufficient data are available to evaluate their prevalence and distributions in the environment [30]. Although their presence in the environment is of considerable concern to public health, no health guidelines or regulations exist to provide a baseline for monitoring these viruses in the environment [31].

PCR methods allow the simultaneous detection of multiple viruses. It can also be used to monitor specific viruses, which may complement the bacterial indicators already used. Some enteric viruses, like the enterovirus, adenovirus, and orthoreovirus groups, are most readily detected with the currently available methods and are recommended for routine monitoring [32]. Another benefit of quantitative real-time PCR is that it permits the evaluation of adenoviral concentrations in environmental samples [30]. In this study, we assessed the prevalence of HAdV and HAV in effluent samples collected from five wastewater treatment plants (WWTPs) located in the Eastern Cape, South Africa. The WWTPs were selected based on their proximity to communities (rural, suburban, and urban areas), treatment technologies, and the lack of research data available for them. To the best of our knowledge, this is the first study of viruses present in the final effluents of WWTPs in the Eastern Cape Province of South Africa.

## Results

### PCR specificity, sensitivity, standard curves, and detection limits

HAdV and HAV were examined with real-time PCR assays. The reactivity of the primers and probes were observed using viral DNA and RNA standards as the templates. Real-time PCR detected HAdV 41 and HAV. The resulting standard curves (HAdV 41, slope −3.53 and Y-intercept 28.34; HAV, slope −3.22 and Y-intercept 36.64) had strong correlation coefficients (r^2^) of 0.99 and 0.98, respectively. The PCR amplification efficiency for the reactions exceeded 92 %. No product was amplified in the negative controls, confirming the absence of PCR carryover contamination.

### Quantification of human enteric viruses in wastewater

**Fig. 1:**
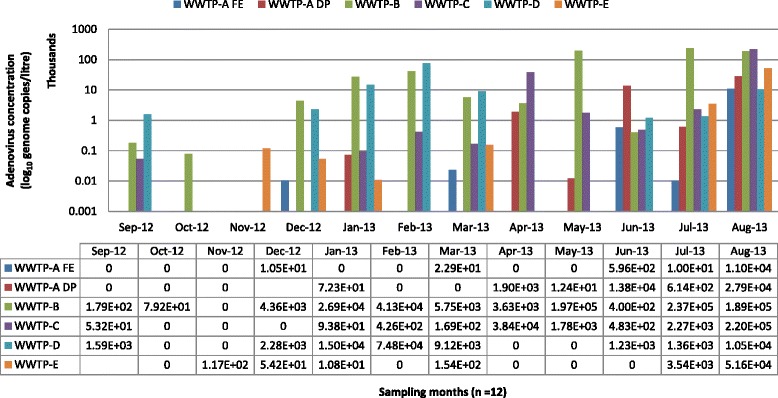
Monthly virus concentrations in effluent samples from five WWTPs. *Characterization of HAdV revealed HAdV 2 in one sample and HAdV 41 in five samples, of the 10 serotypes tested. The detected serotypes belong to specie C and F, respectively. WWTP, wastewater treatment plant; FE, final effluent; DP, discharge point

The results for HAdV detection are summarized in Fig. 1. Adenovirus was detected at all five WWTPs, and in 45 (64 %) of the 70 samples tested. At WWPT-A, two points were sampled, FE and DP. The viral concentrations ranged from 1.05 × 10^1^ to 1.10 × 10^4^ genome/L at FE, with an average concentration of 9.7 × 10^2^ genome/L and a detection rate of 41.7 %. At DP, the average concentration was 4.0 × 10^3^ genome/L, ranging from 1.2 × 10^1^ to 2.8 × 10^4^ genome/L, with a 54.5 % detection rate.

**Fig. 2:**
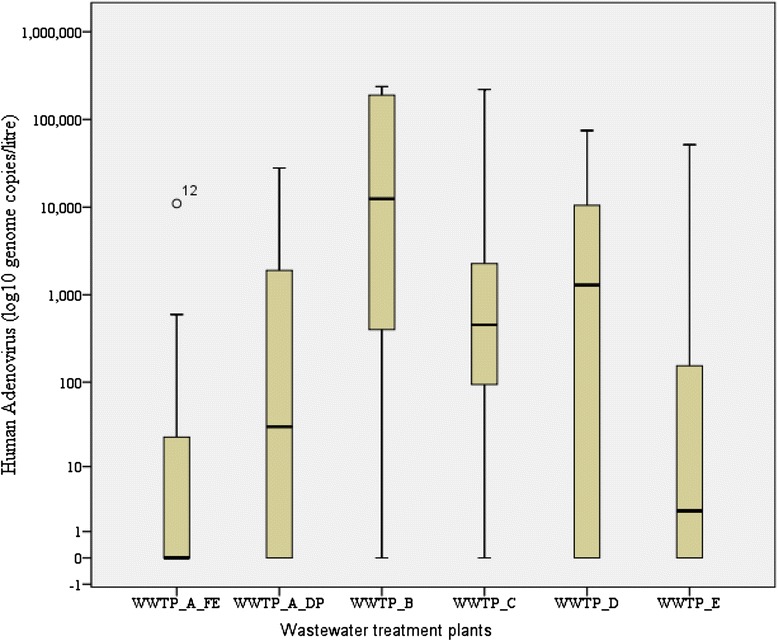
Virus concentrations and distributions at each facility. WWTP, wastewater treatment plant; FE, final effluent; DP, discharge point

At FE, the lowest viral concentration occurred in July 2013 and the highest in August 2013 (Fig. 1). At DP, a low viral concentration was recorded in May 2013 and a high concentration in August 2013 (Fig. 1). Throughout the 12-month sampling period, the HAdV concentration was higher at DP than at FE at WWPT-A. At WWPT-B, the virus was detected in 91.7 % of the samples analyzed, with an average concentration of 5.9 × 10^4^ genome/L, ranging from 7.92 × 10^1^ to 2.37 × 10^5^ genome/L. The lowest and highest viral concentrations were recorded in October 2012 and July 2013, respectively. The average concentration of HAdV at WWPT-C was 2.2 × 10^4^ genome/L, ranging from 5.32 × 10^1^ to 2.20 × 10^5^ genome/L. The lowest concentration was in September 2012 and the highest in August 2013, with a detection rate of 75 %. The average viral concentration at WWPT-D was 9.7 × 10^3^ genome/L, ranging from 1.23 × 10^3^ to 1.05 × 10^4^ genome/L, with a viral detection rate of 66.7 %. The lowest viral concentration was recorded in June 2013 and the highest in August 2014. WWPT-E had an average viral concentration of 4.6 × 10^3^ genome/L, ranging from 1.08 × 10^1^ to 5.16 × 10^4^ genome/L, and a detection rate of 54.5 %. The lowest concentration of virus was in January 2013 and the highest in August 2013 (Fig. 1). The viral detection rates were high during winter and early spring (June–August 2013) and high viral concentrations also occurred within this period. However, in summer (October–February 2013) the viral detection rates and concentrations were low. HAdV was detected at all WWTPs in August 2013 and July 2013, with the highest concentrations at all the WWTPs recorded in August 2013 (Fig. 1). The lowest detection rate for HAdV at a single WWTP occurred in October and November 2012. The highest viral concentrations and detection rates were recorded at WWTP-B, followed by WWTP-C and WWTP-D, whereas WWTP-A had the lowest concentrations and detection rates (Fig. 1). The viral distributions and concentrations varied at all WWTPs (Fig. 2). WWTP-C had a more evenly distributed viral presence in its final effluent than the other treatment plants. The high variability in HAdV in the effluents at WWTP-B, −C, −D, −E, and -A is shown in Fig. 2.

### Adenoviral species and serotypes

Typing the HAdV detected in the effluent samples indicated the presence of two of the four assayed species of adenovirus. The samples analyzed were positive for adenoviral species C and F. Adenoviral species C was positive for serotype 2 and negative for serotypes 1, 5, and 6. Adenoviral species F was positive for serotype 41, whereas serotype 40 was not detected. Five Adenoviral serotype 41 of the species F was detected in five samples (7.1 %), and was the most prevalent serotype, followed by serotype 2 of adenovirus species C (1.4 %). No other species or serotype was detected.

### HAV detection

HAV was not detected in any of the samples collected from four WWTPs. However, it was detected at WWTP-D in 41.7 % of the samples analyzed. However, the viral concentration was ≤ 1 genome copy/L, below the set detection limit.

## Discussion

In this study, we provide conclusive evidence of the presence of HAdV in effluent samples from the East Cape Province, together with its genomic concentrations, but HAV was generally not detected. The total genomic copies of HAdV detected reflect the concentrations of the virus being released into the environment. In this study, HAdV was detected in 64 % of samples and HAV was detected in none. These findings are similar to those of previous studies, which reported HAdV in ≥ 50 % of wastewater and environmental water samples [29, 33–35]. The failure to detect HAV in this study is consistent with previous reports [35, 36], and other studies have shown that HAV is the least detected of the enteric viruses [37]. High concentrations of HAV have been found in sewage samples, with higher concentrations in raw samples than in treated samples [38]. It is possible that HAV was present during the course of the 12-month sampling period at concentrations below our detection limit (10 genome/L). However, HAV was only detected at WWTP-D and at very low concentrations, far below the set detection limit.

High detection rates and viral concentrations of HAdV occurred in winter, between June and August 2013, at all the WWTPs tested. During winter, the treatment efficiency of the WWTPs is reduced because low temperatures negatively affect the process units, which in turn affects the quality of the treated effluent [39, 40]. The detection of HAdV specifically in winter has been reported by Rigotto et al. [41], Haramoto et al. [42], and Haramoto et al. [43]. Furthermore, infections attributable to adenovirus are reported by Modarres and Jam-Afzon [44] to peak in winter, while remaining low in summer [45].

In the present study, we detected no virus at high chlorine concentrations of > 0.20 mg/L (data not shown), whereas at chlorine concentrations ≥ 0.15 mg/L, low concentrations of HAdV were detected, specifically in the WWTP-A and WWTP-E treated effluents. The the other WWTPs showed high detection levels, where effluent quality was poor and chlorine disinfection inefficient. This was evident at WWPT-B and WWPT-C, and WWPT-B recorded the highest concentrations and detection rates of HAdV. Simmons and Xagoraraki [29] and the Water Research Foundation [46] reported that, given the right treatment system and the correct configuration of treatment process, chlorine disinfection will inactivate any HAdV in the effluent. Studies by Thurston-Enriquez et al. [47, 48] demonstrated the inactivation of HAdV 40 with chlorine and chlorine dioxide. They found that the disinfection process is effective at pH 5–8 and temperatures of 5–15 °C, and that contact times < 30 min are sufficient to inactivate HAdV 40. However, they were quick to point out the shielding effects associated with particular matter, which could explain the inefficient disinfection at the WWTPs with high viral loads.

Most of the HAdV serotypes in the effluents were from species C and F. The detection rates for species C and F were 2.9 % and 14.3 %, respectively. A similar study by Van Heerden et al. [16] identified serotypes 2 and 41 in river water receiving wastewater discharge. The incidence of these two serotypes in wastewater effluents has also been reported by Fong et al. [33] and Kuo et al. [7], who detected HAdV 41 more frequently than HAdV 2, which is consistent with our findings. Sibanda and Okoh [17] reported the detection of species C and F HAdV in river water in the Eastern Cape. A study of HAdV in sewage from the Taiwan area reported the presence of both adenoviral species C and F, with a preponderance of HAdV serotype 41 [49]. However, in contrast to the report of Sibanda and Okoh [17], more species C HAdV than species F was detected in river water receiving wastewater discharge in the present study. Respiratory illnesses have been attributed to species C adenoviruses [46], whereas species F is considered one of the major causes of viral gastroenteritis [7]. Species F is reported to cause serious infections in immunocompromised individuals [9], and is often a coinfection in patients with *Human immunodeficiency virus* infections [50].

The high detection rate and high concentrations of HAdV in the final effluents demonstrate the prevalence of the virus in the environment and the disease burden this virus poses in the community. Throughout the months of sampling, the virus was detected regularly at some WWTPs. Wastewater treatment, access to sanitation, and the restoration and rehabilitation of the existing wastewater infrastructure are under discussion at present. With greater droughts occurring globally and the need to move from wastewater treatment to resource recovery, and particularly water recycling, it is extremely important to understand the concentrations of viral pathogens. Real-time PCR, with its high specificity for adenoviruses, is a reliable tool for monitoring viral contamination and pollution in the environment. The use of HAdV as an indicator of fecal contamination is recommended because it is reportedly more stable in the environment and more abundant than other enteric viruses [6, 51]. The frequency of detection of this HAdV in this study supports these views.

## Conclusions

In this study, a 12-month sampling program was conducted at five WWTPs to determine the prevalence of HAdV and HAV in their final effluents. The identification and confirmation of HAdV serotype 2 (species C) and HAdV serotype 41 (species F) as the predominant adenoviral species in this study does not necessarily imply the absence of other serotypes. However, their presence signifies the imminent danger posed to public health by the discharge of poorly treated effluent into the environment because these two adenoviral species have been implicated in clinical illnesses. The presence of viral genomes indicates that the quality of the effluent is low from the perspective of infection risk, although other infectivity assays should be performed to corroborate the potential infectivity of these viruses. To our knowledge, this is the first study to demonstrate the presence and prevalence of HAdV and HAV in the final effluents of WWTPs in the Eastern Cape Province of South Africa.

## Materials and methods

### Sampling sites

For confidentiality, the WWTPs are listed as WWTP-A, WWTP-B, WWTP-C, WWTP-D, and WWTP-E. WWTP-A operates an activated sludge system with a design capacity of about 8 ML/day; WWTP-B has a design capacity of 5 ML/Day and operates a biofilter/PETRO® (pond enhanced treatment and operation) treatment system; WWTP-C operates an activated sludge system with a design capacity of 40 ML/day; WWTP-D operates both a biofilter and an activated sludge system, with a design capacity of 12 ML/day; and WWTP-E has a design capacity of 1.8 ML/day and operates a biofilter system. All the WWTPs use chlorine disinfection.

### Sample collection

Samples were collected from the five WWTPs from September 2012 to August 2013, at two sampling points for WWTP-A: the final effluent point (FE) just after chlorination and the discharge point (DP), immediately before the wastewater is discharged into the river. WWTP-B, WWTP-C, WWTP-D, and WWTP-E were sampled at FE only, their DP was inaccessible. Effluent samples were collected in sterile 1.7-L Nalgene bottles containing sodium thiosulfate to dechlorinate the samples. A cooler box was used to store all the samples and transport them to the laboratory for processing within 2 h. The effluent samples were collected as part of the routine surveillance of enteric viruses at each WWTP. The samples were collected once a month at each WWTP (*n* = 12). No samples were collected at WWTP-A (DP) in December 2012 or at WWPT-E in September 2012 because climatic conditions were unfavorable, so a total of 70 samples were processed.

### Concentration of water samples for viral detection

The viruses in the effluent samples were concentrated with the adsorption–elution method, as described by Haramoto et al. [52], with some modifications. A sample (5 mL) of 250 mM AlCl_3_ was passed through a Millipore type HA filter held for 5 min (0.45-μm pore size and 47-mm diameter) to generate a cation (Al^3+^)-coated filter, which was attached to a 250-mL Millipore sterile filtration system on t3-place filtration manifold. A 1.25-L sample of effluent was passed through the filter, and 200 mL of 0.5 mM H_2_SO_4_ was then filtered through the membrane. The viral particles were eluted into a Petri dish with 10 mL of 1 mM NaOH. The eluates were placed in Centriprep™ Centrifugal Filter Units with Ultracel-50 membranes, containing 0.1 mL of 50 mM H_2_SO_4_ and 0.1 mL of 100 × Tris–EDTA (TE) buffer to neutralize them before further concentration. The Centriprep™ YM-50 ultra-filtration device (Millipore) was centrifuged to produce a final volume of approximately 700 μL. In exceptional cases, when the eluate was turbid, the centrifugation time was increased and the clogged membrane was cleared with sterile forceps. The concentrated samples were stored at −80 °C until use.

### Control strains

The prototype strains of HAdV (ATCC VR-931, strain Dugan) and HAV (ATCC VR-1357, strain PA21) used in this work were obtained from the American Type Culture Collection (ATCC, Rockville, MD).

### Extraction of viral nucleic acids

Viral nucleic acids were extracted from 200 μL of the concentrated effluent samples with Quick-gDNA™ MiniPrep and a Zymo Viral RNA Extraction Kit (Zymo Research Corporation, 17062 Murphy Ave. Irvine, CA 92614, U.S.A) using the spin column technique, according to the manufacturer’s instructions. All samples were tested for the presence of HAdV and HAV nucleic acids with real-time PCR.

### Quantification of viral genomes with real-time PCR

HAdV was quantified with quantitative PCR (qPCR) in a one-step reaction in a 96-well plate. The wells were loaded with 20 μL of reaction buffer containing 12.5 μL of 2 × TaqMan Universal PCR Master Mix (Applied Biosystems), 400 nM forward primer, 400 nM reverse primer, 250 nM TaqMan probe, and PCR-grade water. Aliquots (5 μL) of the sample DNAs were then added and mixed, in total reaction volumes of 25 μL. Amplification was performed on a StepOnePlus™ Real-time PCR System thermal cycler (Applied Biosystems) with preliminary denaturation and the following cycling parameters: 15 min at 95 °C to activate the *Taq* DNA polymerase, followed by 45 cycles of denaturation at 95 °C for 10 s, annealing at 55 °C for 30 s, and extension at 72 °C for 20 s. The primers and probes used for real-time PCR are shown in Table 1. The HAdV strain Tak (ATCC VR-930) was used as the positive control.

**Table 1:** Primers and probes for real-time RT–PCR and qPCR

Enteric virus	Primers and Labelled TaqMan Probe	Reference
Hepatitis A virus	HAV68 (F): 5′-TCA CCG CCG TTT GCC TAG-3′	[53, 54]
	HAV240 (R): 5′-GGA GAG CCC TGG AAG AAA G-3′	
	HAV150 (P): 5′-FAM-CCT GAA CCT GCA GGA ATT AA- MGBNFQ-3′	
Adenovirus	JTVX(F) 5′-GGACGCCTCGGAGTACCTGAG-3′	[30]
	JTVX(R) 5′-ACIGTGGGGTTTCTGAACTTGTT-3′	
	JTVX(P) 5′-FAM-CTGGTGCAGTTCGCCCGTGCCA-MGBFQ-3′	

*F* forward/sense, *R* reverse/antisense, *P* probe, *FAM* 6-carboxyfluorescein (reporter dye), *MGBNFQ* minor groove binder/nonfluorescent quencher

The HAV RNA virus was quantified with a two-step protocol, in which the RNA was first transcribed into cDNA in a separate reverse-transcription step. Briefly, 10 μL of template RNA, 1 μL of 100 μM random hexamer primer, 1 μL of 100 mM dNTP mix, 2.5 μL diethylpyrocarbonate (DEPC)-treated water, 4 μL of 5 × RT buffer, 0.5 μL of 40 U/μL RiboLock RNase Inhibitor, and 1 μL of 200 U/μL RevertAid Premium Reverse Transcriptase (Fermentas Life Sciences) were added in the order indicated to a 0.5-mL PCR tube on ice, mixed by vortexing briefly, and centrifuged (15,000 × g). The tube was incubated at 25 °C for 10 min, and then for 30 min at 60 °C. The reaction was terminated by heating at 85 °C for 5 min. An aliquot of 5 μL of the resultant cDNA was used as the template for a real-time qPCR reaction containing reagents in the same proportions as were used to amplify HAdV. HAV strain PA21 (ATCC VR-1357) was used as the positive control. Fluorescence data were collected at the end of the annealing step.

### Identification of adenoviral species and serotypes

Serotype-specific PCR assays with the PCR conditions described by Metzgar et al. [55] for species B–E, and the reaction described by Tiemessen and Nel for species F [56] were used to identify the adenoviral serotypes. The HAdV serotypes were determined in all the samples analyzed. The primers used are shown in Table 2. For quality assurance, specific adenoviral strains were used as controls.

**Table 2:** Primers for the detection of adenoviral serotypes

Species	Serotype	Primer	Sequence (5′ to 3′)	Target region
B	Ad3	Ad3F	GGTAGAGATGCTGTTGCAGGA	Ad3 hexon
		Ad3R	CCCATCCATTAGTGTCATCGGT	
	Ad7	Ad7F	GGAAAGACATTACTGCAGACA	Ad7 hexon
		Ad7R	AATTTCAGGCGAAAAAGCGTCA	
	Ad21	Ad21F	GAAATTACAGACGGCGAAGCC	Ad21 hexon
		Ad21R	AACCTGCTGGTTTTGCGGTTG	
C		AdCF	TGCTTGCGCTHAAAATGGGCA	AdC fiber
	Ad1	Ad1R	CGAGTATAAGACGCCTATTTACA	Ad1 fiber
	Ad2	Ad2R	CGCTAAGAGCGCCGCTAGTA	Ad2 fiber
	Ad5	Ad5R	ATGCAAAGGAGCCCCGTAC	Ad5 fiber
	Ad6	Ad6R	CTTGCAGTCTTTATCTGAAGCA	Ad6 fiber
E	Ad4	Adeno4.U3	CAAGGACTACCAGGCCGTCA	Ad4 hexon
		Adeno4.L1	TTAGCATAGAGCATGTTCTGGC	
F		AdF1	ACTTAATGCTGACACGGGCAC	Fiber
	Ad40	K402	CAC TTA ATG CTG ACA CG	
	Ad41	K403	ACT GGA TAG AGC TAG CG	

### Prevention of PCR carryover contamination

All standard precautions were taken to prevent PCR contamination, with adherence to strict laboratory practices. The pre-PCR manipulations (DNA isolation and PCR set-up) were performed in a clean room that was physically isolated from the real-time PCR machine and the post-PCR processing area. Dedicated pipettes and reagents were used at each location. Negative controls were run with all assays, and no indications of contamination were detected. The DNA used to generate the standard curves was prepared in a separate room.

### Sensitivity and specificity studies

To validate the real-time PCR assays before their application to the effluent samples, the detection limit and amplification efficiency of each reaction were determined as described by Simmons and Xagoraraki [29]. The sensitivity of our real-time PCR assay was evaluated with the nucleic acid from a stock culture of HAV and with HAdV DNA from a serial seven-fold dilution of a genomic extract. A detection limit of 10 copies of target DNA per reaction was set for all PCR assays.

### Standard curve construction

Standard curves were generated to quantify the sensitivity of the assays using stocks of HAdV and HAV. The extracted DNA and RNA were prepared and their concentrations determined spectrophotometrically with a Qubit® 1.0 Fluorometer (Life Technologies), according to the manufacturer’s instructions. The viral DNA and RNA were serially diluted seven-fold in nuclease-free water to generate the standard curves. All the standard curve reactions were run in triplicate.
